# Patterns of patient and healthcare provider viewpoints regarding participation in HIV cure-related clinical trials. Findings from a multicentre French survey using Q methodology (ANRS-APSEC)

**DOI:** 10.1371/journal.pone.0187489

**Published:** 2017-11-02

**Authors:** Christel Protière, Bruno Spire, Marion Mora, Isabelle Poizot-Martin, Marie Préau, Marjolaine Doumergue, Philippe Morlat, David Zucman, Cécile Goujard, François Raffi, Olivier Lambotte, Marie Suzan-Monti

**Affiliations:** 1 Aix Marseille Univ, INSERM, IRD, SESSTIM, Sciences Economiques & Sociales de la Santé & Traitement de l’Information Médicale, Marseille, France; 2 ORS PACA, Observatoire régional de la santé Provence-Alpes-Côte d’Azur, Marseille, France; 3 Aix-Marseille Univ, CISIH, APHM, Hôpital Sainte-Marguerite, Marseille, France; 4 GRePS Lyon 2 Université, Bron, France; 5 Service de Médecine Interne et Maladies Infectieuses, Hôpital Saint André, CHU de Bordeaux; Université de Bordeaux; INSERM U 1219; Bordeaux, France; 6 Hôpital Foch, service de médecine interne, Suresnes, France; 7 Assistance Publique—Hôpitaux de Paris, Hôpital Bicêtre, Service de Médecine Interne et Immunologie clinique, Le Kremlin-Bicêtre, France; 8 Service de Santé publique, AP-HP et INSERM 1018, Centre de recherche en Épidémiologie et Santé des Populations (CESP), Université Paris-Sud, Villejuif, France; 9 University Hospital, Department of Infectious Diseases, Nantes, France; 10 INSERM, U1184, Immunology of Viral Infections and Autoimmune Diseases, Le Kremlin-Bicêtre, France; 11 Université Paris Sud, UMR 1184, Le Kremlin-Bicêtre, France; 12 CEA, DSV/iMETI, IDMIT, Fontenay-aux-Roses, France; Fundacao Oswaldo Cruz, BRAZIL

## Abstract

**Context:**

Despite huge advances in the fight against HIV concerning diagnosis, clinical efficacy of antiretroviral treatments (ART), patient survival and quality of life, there is still no cure. Recent developments in HIV cure research have opened the way for clinical trials which could lead to a temporary or definitive end to ART. However, ethical questions exist about related trial-participation risks. The main goal of the ANRS-APSEC survey was, using Q-methodology, to investigate the viewpoints of people living with HIV (PLWH) and HIV healthcare providers (HHP) regarding motivations for and barriers to participation in HIV Cure-related clinical trials (HCRCT).

**Materials and methods:**

Thirty-three statements were defined encompassing seven dimensions: treatment and follow-up; risks; benefits; patient-physician relationship; beliefs and attitudes; information; target population. Forty-one PLWH and 41 HHP from five French HIV services were asked to rank-order the statements.

**Results:**

Five main viewpoints were elicited from “the most motivated” to “the most reluctant” vis-à-vis HCRCT participation. All placed importance on the wish to participate in HIV research. This result is in line with the HIV-specific culture of joint mobilization. For some viewpoints, the motivation to participate in/propose HCRCT was primarily conditioned by side-effects and/or by constraints, which overall were more accepted by PLWH than HHP. Some viewpoints placed particular importance on HCRCT recruitment strategies. Finally, some expressed a high acceptance of risks and constraints but emphasized the need for information.

**Conclusion:**

HIV cure research clinical trials (HCRCT) constitute a risky yet unavoidable step towards the goal of finding a cure. To improve future HCRCT and informed consent designs, based on PLWH and HHP preferences and expectations, we need greater knowledge about how these populations perceive the risks and the benefits of HCRCT. Our results confirmed the importance of careful, studied HCRCT design, management and communication, to ensure PLWH and HHP acceptability and convergence of their expectations.

## Introduction

Since the advent of HIV and the first AZT treatment [1], huge advances have been made in terms of detection of the virus, clinical efficacy, patient survival and quality of life (QoL) [2–5]. Current antiretroviral treatments (ART) are well-tolerated and cause fewer side-effects, but do not cure the virus, mainly because of viral persistence in cell reservoirs [6]. People living with HIV (PLWH) have to take treatment at least once a day, every day for the rest of their lives. In addition to the difficulty of treatment adherence over such a long period, daily medicine is a constant reminder to the patient of the disease. Furthermore, it impacts QoL and is associated with long-term comorbidities. Moreover, despite HIV becoming a manageable chronic illness, risky behaviours [7] and stigmatization [8,9] are still matters of concern. In addition, ART have serious economic consequences on public health [10].

Recent developments in HIV-cure research have opened the way for HIV Cure-related clinical trials (HCRCT) which could lead to a temporary or definitive end to ART [11–15]. The greater sense of hope for a cure is counterbalanced by ethical questions regarding HCRCT-participation risks [16–18] for PLWH and HIV healthcare providers (HHP) alike. Future HCRCT will target controlled patients with “normal” lives [19]. Participating in HCRCT may have consequences on daily lives because of treatment administration and follow-up procedures, and on health because of potential treatment side-effects and ART interruption. More specifically, treatment interruption could lead to increased viral load and increased HIV transmission risk, with consequences for individual and general public health (a summary of the main results of HCRCT is provided in Table 1 in Dubé et al. [20]). This might impact negatively on recruitment for HCRCT, especially given their limited direct benefit for participants [21,22]. Furthermore, PLWH might misunderstand that the objective of such clinical research is to produce knowledge and not necessarily to produce any direct benefit [20,23]. Despite these risks, the possibility of moving from an incurable to curable disease may encourage participation by treated PLWH, and more engagement by untreated PLWH in testing and care [24].

The multi-centre ANRS-APSEC project (Acceptabilité, Attente et Préférence des Patients et des Soignants vis-à-vis d’Essais Cliniques de guérison du VIH) formed part of the IAS “Towards an HIV cure” initiative which promotes multidisciplinary research for a safe, affordable and scalable cure [25,26]. The second step of this project, aimed to provide evidence about stakeholders’ viewpoints regarding motivations for and barriers to their participation in HCRCT. To reach this goal, we employed Q methodology which explores subjective viewpoints [27]. It not only helps to elicit the structure of the viewpoint regarding the most important dimensions in stakeholders’ decisions to participate, but also the importance of these dimensions in relation to each other. Because of the importance of the patient-physician relationship [7,28], which is reinforced by the chronic nature of the disease, and because stakeholders occupying different positions in the process may have different viewpoints and different priorities about HCRCT [20,29], we included PLWH eligible for HCRCT and different types of HHP. Previous surveys [7,17,21,24] mainly focused their investigation on PLWH only, with the exception of one qualitative survey [20] which included PLWH and HHP. However, the latter did not specifically target PLWH who met the probable clinical criteria for inclusion in future HCRCT [29]. Neither did it target certain HHP (including nurses, research technicians, etc.) not directly involved in HIV cure research.

## Materials and methods

**Fig 1:**
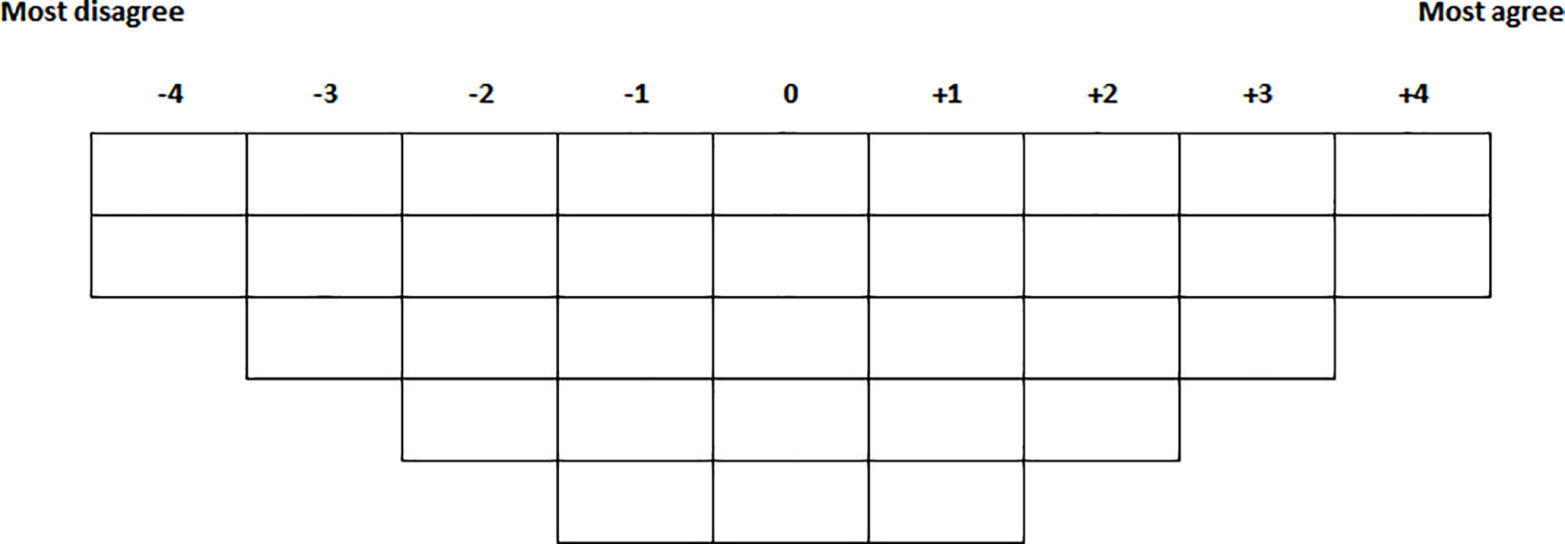
Grid distribution.

Q-methodology combines qualitative and quantitative approaches [30–33] and has already been applied in several health-related studies [34–38]. In Q studies, respondents rank-order a set of statements about a specific topic on a grid which is labelled according to one subjective dimension, for example ‘agreement/disagreement’ (Fig 1). This rank ordering, called a Q-sort, is obtained for each respondent, based on a common set of propositions [39].

Shared *viewpoints* were obtained by a factor analysis performed on the Q-sorts: each factor represents a viewpoint shared by a group of respondents who rank-order a set of statements in a similar fashion. If different individuals have different viewpoints on the topic, their Q-sorts will not correlate. If significant clusters of correlation exist, they can be factorized, and individuals are assigned to a particular factor [40]. Each viewpoint is described by a distinct ordering of statements. Subsequently, interpretation does not account for statements in isolation, but collectively and relative to the position of the other statements.

### Definition of the statements set (Q-set)

To identify a comprehensive list of statements reflecting opinions about HCRCT participation, three sources were used. A literature review was first implemented to design the focus group guides. Six focus-groups (three PLWH (n = 21) and three HHP (n = 30)) in three French HIV care units (Hôpital Bicêtre, Le Kremlin Bicêtre; CHU Mondor, Créteil; CHU Hôtel-Dieu, Nantes) were then conducted by three sociopsychologists (MD, MP and MM) who obtained written informed consent from participants before proceeding [41]. Finally, semi-structured individual interviews with three PLWH and six HHP helped complete the set of statements.

A first set of 56 statements was extracted by two researchers (CP and MD) from the transcripts of the focus groups and the individual interviews. After discussion with clinicians participating in the study, 34 statements were retained. Pilot testing of the these statements, conducted by two researchers (CP and MM) with 7 HHP and PLWH (CHU Sainte Marguerite in Marseille and PLWH from the French NGO AIDES) led to minor rewording and the deletion of one statement. The wording of the final set of 33 statements was adapted to both two surveyed populations (Table 1 and S1 Table for details of the PLWH and HHP statements, respectively).

**Table 1:** Description of persons living with HIV statements.

Treatment and follow-up	s1	In order for me to participate, the monitoring of the treatment-free phase must be adapted fully to each patient (tailor-made)
	s2	In order for me to participate, the follow-up during the treatment-free phase must be regular and closely monitored (from once a week to once a day).
	s4	I would not participate if the medical follow-up of the treatment-free phase were too restrictive
	s5	I would refuse to participate if the innovative treatment were to last more than 6 months
	s6	I would participate only if the innovative treatment was administered on an outpatient basis and did not require hospitalization
Risks, Adverse effects and QOL	s7	I would not participate because of the possible increased risk of HIV transmission during the treatment-free phase
	s8	I would not participate if the adverse effects of the innovative treatment were too acute (loss of autonomy or bedridden for 4 to 8 days per month)
	s9	I would not participate if the innovative treatment were to affect vital organs
	s10	In order for me to participate, the innovative treatment must not entail anything irreversible in terms of adverse effects
	s11	If the adverse effects were to last more than 5 days after taking the innovative treatment I would refuse to participate
	s12	In order for me to participate, it is fundamental that my lifestyle does not change
Physician-patient Relationship	s3	In order for me to participate, it is fundamental that my current physician for HIV supervise the three phases of the trial
	s13	In order for me to participate, it is fundamental that my current physician for HIV believe in it
	s14	In order for me to participate, a medical contact must always be available by phone
Beliefs and Attitudes	s15	I don’t believe in this type of trial.
	s17	I believe that participating in a clinical trial guarantees better treatment
	s18	I think that it would be better to invest in access to ARV treatment for everyone
	s19	I think that it would be better to invest in prevention
Benefits	s20	Financial compensation could entice me to participate
	s21	I would participate in this trial if the treatment-free phase did not last more than 6 months
	s22	It is important to participate in HIV research
	s23	It is important that medical advances for future generations be made possible
	s24	One motivation to participate in this trial would be that one could forget about the disease
	s25	Proposing a clinical (research) trial with no direct benefit for the patient has no sense
	s26	Participating in this trial could be a way to avoid the long-term consequences of ARV treatment.
Information	s16	There is too much uncertainty about the adverse effects for me to participate
	s27	Having regular feedback from participating patients would motivate me to participate
	s28	Being provided with regular information by physicians about the trial’s results would motivate me to participate.
	s29	In order for me to participate, it is necessary that I have clear information about the treatments.
Target Population	s30	This trial is more suitable for people diagnosed more than ten years ago.
	s31	This trial should primarily focus on those who have participated in only a few trials to date
	s32	This trial is more suitable for people who find it difficult to take ARV treatment on a daily basis
	s33	This trial is more suitable for people who do not work

The final Q-set covered the range of issues at stake, which were classified into seven dimensions: 1/ Treatment modalities and follow-up (5 statements), 2/ Risk, adverse effects and quality of life (6 statements), 3/ Patient-physician relationship (3 statements), 4/ Belief and attitudes (4 statements), 5/ Benefits (7 statements), 6/ Information (4 statements), and 7/ Target population (4 statements).

### Respondents (P-set)

In Q-studies, selection of the respondents is purposive as in qualitative techniques. With respect to the size of the P set, Brown suggested that 40–60 respondents are more than likely adequate [33,38,42]. Because of possible differences in viewpoints between PLHW and HHP, we planned to recruit 40 respondents from each group to perform data analysis at the population level. Respondent recruitment and questionnaire administration occurred between June and July 2015 in five French HIV services with a history of HIV clinical trial participation. Some, but not all, were involved in HIV cure research, (Hôpital Bicêtre, Le Kremlin Bicêtre; CHU de Bordeaux, Bordeaux; CHU Sainte Marguerite, Marseille; CHU Hôtel-Dieu, Nantes; Hôpital Foch, Suresnes). PLWH were recruited the day of their follow-up consultation and were provided details about the study by the clinical research technician of each unit and through an information letter. HHP were also informed through an information letter. All respondents were assured of the confidentiality and anonymity of their answers. Written informed consent was obtained from:

✓ 41 PLWH who met the following eligibility criteria: stable ART treatment (>6 months), sustained virological suppression and a CD4 cell count >500 CD4/mm^3^.
✓ 41 HHP comprising physicians, nurses and clinical research technicians.

### The Q-sort exercise

Before proceeding with the Q-sort exercise, a three-step structure of hypothetical future HCRCT was presented to the respondents. This description was designed with the collaboration of participating clinicians, and reflected the uncertainty related to side-effects and to cure trials themselves as they are currently hypothetical.

1. **Intensification of ART treatment** (3–6 months);
2. **Administration of the innovative treatment** to decrease HIV reservoirs in the organism and/or increase immune defences against HIV. Some adverse effects were predictable and follow-up would be intensified;
3. **Discontinuation of ART treatment** to verify the efficacy of the innovative strategy, i.e. no more virus replication. This would be assessed by regular viral load tests.

The Q-sort was administered face to face, by trained independent interviewers and by one researcher (CP), using a dedicated online Q-sort tool (FlashQ: http://www.hackert.biz/flashq/home/). The online administration enabled direct anonymization of the data by the attribution of an anonymous identification number before proceeding.

Q-sorts required participants to order and prioritize each statement in relation to every other statement according to the following specific instruction: ‘*Our aim is to know your point of view regarding future HIV cure trials and to identify what would motivate or hamper your participation in* (PLWH)*/your decision to propose* (HHP) */ such trials’*.

First, respondents were asked to sort the statements, presented randomly, into three piles: agree, disagree and neutral. They were then guided through the following process: the two statements they agreed with the most were placed under (+4) while the two they disagreed with the most were placed under (-4), and so on, working towards the centre of the grid, until all statements were placed (Fig 1). Once the Q-sort was complete, respondents were invited to examine and adjust it if they wished. They were then asked to provide open-ended comments to explain their rationale for the choice of the four statements ranked at the extremes.

After completing the ranking, respondents were asked for some additional information: gender, sexual orientation, experience with HIV and willingness to propose/participate in HCRT if available.

### Analysis and interpretations

The Q-sorts were analysed using a dedicated software package PQMethod 2.35 (http://schmolck.userweb.mwn.de/qmethod/downpqwin.htm). The first step in Q-factor analysis (here a principal component factor analysis followed by a varimax rotation) is to calculate a correlation matrix, which presents the degree of similarity between individuals’ different Q-sorts. The objective of the factor analysis was to identify a limited number of similar ways in which respondents completed their Q-sorts. For each viewpoint (factor) obtained, an idealized Q-sort was calculated: Q-sorts with higher loadings were given more weight in the idealized Q-sort since they better exemplified the viewpoint [36,42]. This idealised Q-sort represented the fictive Q-sort of a person correlated at 100 percent with that viewpoint.

Interpretation was not performed statement by statement, but rather aimed at presenting a holistic interpretation of each viewpoint. Special attention was paid to certain statements including: ‘Consensus statements’ (scores not statistically different between any pair of viewpoints, p<0.5) which represented statements with which all viewpoints (dis)agreed; ‘extreme statements’ which were placed at the grid poles (+/-4, +/-3) and their associated comments; ‘distinguishing statements’ (a score in one idealised Q-sort statistically different from those in the other idealised Q-sorts); and statements that were ranked higher and lower in a given idealised Q-sort than in each of the other idealised Q-sorts [27].

Hereafter, rankings of the statements are presented in abbreviated form, for instance (s13, +4) means that statement 13 was ranked in the +4 position (agreed the most) and (s15, -4) means that statement 15 was ranked in the -4 position (disagreed the most) for a given viewpoint. A statement in bold means that it was “distinguished”, and statements ranked highest or lowest by one viewpoint were marked by a ‘+’ or a ‘**-**’, respectively. Statements and respondents’ comments were translated from French and quotes are provided here to illustrate and clarify interpretations (respondent type is indicated: PLWH or HHP).

Two analyses were performed, the first on the whole sample, the second on each population. We then performed a second order analysis to choose the strategy to retain. It showed that the first PLWH viewpoint (PLWH_VP1) was correlated with two other viewpoints (PLWH-VP4 and HHP-VP4). This means we would have lost the specificity of this viewpoint if we had performed the analysis on the whole sample. Moreover, the third HHP viewpoint (HHP_VP3) was not related to any other viewpoint (S1 Fig). Accordingly, we retained the analysis by population strategy.

### Ethics

The study was approved by CCTIRS (Advisory Committee on Information processing in Material Research in the field of Health) and CNIL, the French data protection authority (approval number DR-2016-322).

## Results

### Respondents’ characteristics

The PLWH sample (n = 41) comprised a higher proportion of men (80%) and of homosexuals (54%) and bisexuals (10%) to reflect the proportions of registered PLWH in France, whereas the HHP sample (n = 41) comprised a higher proportion of women (66%) and of heterosexuals (90%). Median (IQR) age was 49 (41–53) years old for PLWH and 47 (38–53) years old for HHP. Median duration of HIV experience was 14 (9–21) years for PLWH and 15 (6–20) years for HHP. A large majority of the respondents were motivated to participate in HCRCT: 63% of PLWH and 59% of HHP declared they would certainly participate in HCRT if available, while 34% of PLWH and of HHP said perhaps.

**Fig 2:**
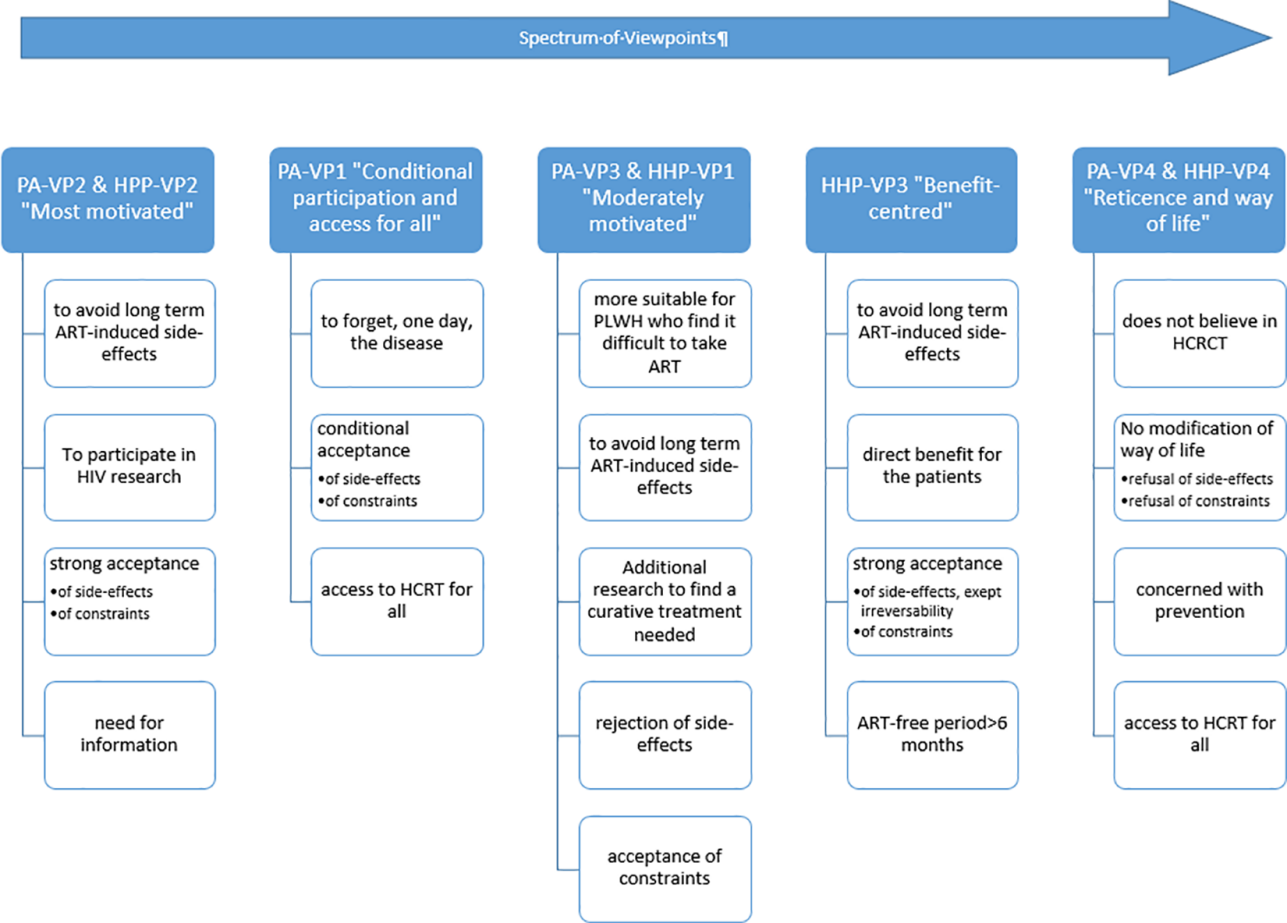
Summarized description of viewpoints.

The factor analysis resulted in a four-factor solution for each population, explaining 62% and 61% of the total variance expressed by PLWH and by HHP, respectively (S2 Table). For each population, a gradient of participation was expressed (Fig 2). Below, we first describe the consensus statements for each population, then the viewpoints.

### Consensus statements

For both PLWH and HHP the frontier column was close to ‘-1’, i.e. respondents expressed disagreement between ‘-4 and -1’ and agreement between ‘0 and +4’.

#### Consensus among PLWH

Seven consensus statements were obtained for the PLWH, concerning four of the seven dimensions investigated.

Altruistic benefits: the importance of participating in HIV research (*s22*, +3, +4, +3, +3) and ensuring that medical advances for future generations be made possible (*s23*, +2, +3, +3, +4), were strong common motivations for participating in HCRCT. Because these two statements were placed at one extremity of the grid, attention should be paid to statements placed at the +2 column in order to characterise the viewpoints.

There was also consensus for the three statements concerning the patient-physician relationship dimension. More specifically, there was strong agreement that one’s current HIV physician needed to believe in the trial in order for PLWH to participate (*s13*, +4, +3, +2, +3), and some degree of agreement that the trial would be more acceptable if patients were supervised by their current HIV physician (*s3*, 0, +2, 0, +2). On the contrary, the availability of a medical contact by phone was not considered a priority (*s14*, 0, +1, -1, -1).

A >6-month duration on the innovative treatment was not seen as a barrier to participation (*s5*, -2, -2, -2, -2). Finally, being provided with regular information by physicians about the trial’s results was a common motivation (*s28*, +1, +2, +2, +3).

#### Consensus among HHP

There were four consensus statements for HHP, each for a different dimension.

Two were common to PLWH statements: the importance of participating in HIV research (*s22*, +1, +3, +3, +1) and the necessity that the PLWH’s current HIV physician believed in the trial in order to propose it (*s13*, +2, +2, +2, +2).

The two others were specific to HHP: participation in a clinical trial did not guarantee better care for patients (*s17*, 0, -1, -1, -1), and HCRCT should not target patients who have previously participated in only a few clinical trials (*s31*, -4, -3, -4, -2).

### Viewpoints specific to only one population

Two of the eight viewpoints (PLWH-VP1 and HHP-VP3), were called “specific” as they were not uniquely associated with another viewpoint according to the second order analysis (S1 Fig).

#### Specific PLWH viewpoint: PLWH-VP1 ‘Conditional participation and access for all to HCRCT’

For this viewpoint, which represented 17% of the total variance, participation in HCRCT would be motivated by the possibility to, one day, forget about the disease (s24+, +2).

PLWH *“Because taking drugs is quite constraining*. *We know they help us to live normally but when you travel*, *you must consider everything*, *such as anticipating the time difference for example*, *and it makes you think about the disease”*

This possibility could be considered a direct benefit to PLWH. The need for direct benefits is supported by this viewpoint (**s25+**, +1), which contrasts with the refusal of financial compensation as a motivation to participate (**s20**, -3) and disagreement with the belief that clinical trials guarantee better care (**s17-**, -2).

Two main points characterized this viewpoint. First the acceptance of some side-effects (**s8**, -1; s11, -1) including possible increased HIV transmission risk during the period without ART (s7+, -1). However, the irreversibility of side-effects (**s10**, +3) or side-effects affecting vital organs (s9, +4) represented a threshold for refusal to participate. Constraints arising from HCRT participation were also accepted on the condition that there would be regular and closely monitored follow-up during the period without ART (s2+, +2) and that the innovative treatment would be administered on an outpatient basis (s6+, +2). As one patient commented, “*Hospitalisation could remind you of the disease*”.

Second, this viewpoint strongly reflected the desire for access to HCRCT for the highest number of PLWH (including themselves), and expressed substantial disagreement with any selection of the target population (**s30-**, -4; s32-, -3; s31, -2; s33, -1).

PLWH “*Because trials are for everyone*. *Everyone has the right to be cured*”.

PLWH “*Being seropositive for 4 years*, *I found the question unsuitable*”.

Finally, for this viewpoint, clear information about the innovative treatment was important (s29, 3), to “*better enable oneself to psychologically prepare*”.

#### Specific HHP viewpoint: HHP-VP3 ‘*Benefits-centred’*

For this viewpoint, which represented 13% of the total variance expressed by HHP, participation in HCRCT was considered a means to avoid long-term ART-induced side-effects (s26, +3). This viewpoint, like PLWH-VP1, considered that proposing a trial without any direct benefit for the patient has no sense (s25+, +4), even though altruistic benefits were also emphasised (*s22*, +3; s23, +2).

HHP “*Knowing that it’s a therapeutic trial*, *and not a cohort or physio-pathological survey*, *it would be unethical to propose a survey without any direct benefit for the patient*”.

The expected benefits—requiring a greater than 6 months ART-free period duration (**s21-**, **-3**)—justified the acceptance of a certain number of constraints and side-effects (s11, -2; s7, -1; s9, +1), including intense ones (**s8-**, **0**), but excluding potential irreversibility (s10, +4).

HHP “*The risk cannot be greater than the benefit for the patient*”

HHP “*Irreversible sequelae would imply the need for other treatments for the patient*, *and as a result*, *the trial would present more drawbacks than advantages*”

HHP “*It presents a lot of upstream constraints for the patient*, *for such a short period* [less than 6 months]”

Moreover, this viewpoint considered that patients’ ways of life would inevitably be modified (**s12-**, **-3**), principally because of follow-up during the treatment-free period (s2+, +2). Any possibility of personalizing this follow-up would be excluded (**s1-**, **-4**) because it was perceived as a risk for the patient.

HHP “*Patients’ ways of life will inevitably be modified*, *since their participation will mean more medical appointments*, *even day hospitalization*, *and potentially adverse effects with an impact on their quality of life*”

HHP “*On the flipside*, *it* [the follow-up] *would need to be well defined in the protocol and strictly observed*”

This viewpoint contrasted with all others, except PLWH-VP4, as it considered that it is better to invest in access to ART for all PLWH (**s18+**, +2). Little consideration was given to the patient selection, except for the strong disagreement that any priority should be given to patients who had already participated in only a few clinical trials (*s31*, -4).

### Shared viewpoints between PLWH and HHP

The following three pairs of viewpoints are presented, highlighting both their common and differing traits between PLWH and HHP, in order to emphasize the specificity of each population.

#### ‘*Reticence and way of life’* viewpoints PLWH-VP4 and HHP-VP4

These two viewpoints each represented 14% of the variability.

Although they mostly agreed that it is important that medical advances for future generations be made possible (s23+, PLWH/**HHP +4),** they reflected less belief in HCRCT than the six other viewpoints (**s15+**, PLWH/HHP **-1**).

For the PLWH viewpoint, medical advances seemed to be linked with a priority given to prevention (PLWH: **s19+**, **+2**), illustrated by the following comment: “*the objective is to avoid new contamination*”.

The HHP viewpoint was more motivated by avoiding any risk of transmission associated with HCRCT (HHP: s7+, +2), and considered that proposing a trial without any direct benefit has no sense (HHP: **s25**, +2). One prior HHP experience emphasized this point: “*I felt uncomfortable with my patient relationship when announcing the viral load during the period without any ART treatment*”. Moreover, this HHP viewpoint did not consider, unlike the other HHP viewpoints, that HCRCT would be an opportunity to reduce the long term side-effects of ART (HHP: **s26-**, **-1**).

The second characteristic of these two viewpoints was the rejection of constraints on participating patients that would bring about a modification of the patients’ way of life [HHP: (s1+, +1; **s2-**, **-3**; **s4+**, **+2**; **s6+**, **+3**; s8+, +4; s10, +3; s12+, +3); PLWH: (s1, +1; s6, +1, s4+, +2; s12+, +2)].

HHP were more concerned than PLWH by side-effects, but mainly because of the associated loss of patient autonomy, as illustrated by an HHP comment: “*today*, *for PLWH who are well controlled by ART*, *life is almost normal for most of them*. *This is why I don’t see the value in proposing a disabling treatment*”.

Finally these two viewpoints strongly rejected monetary compensation (**s20-,** PLWH/**HHP**: **-4**) and, like PLWH-VP1, selection of the target population (PLWH: s30, -1; s31, -1; s32, -2; **s33-**, **-3;** HHP: s33-, -3; *s31*, -2; s32, -2), with the exception of patients diagnosed for more than ten years for the HHP viewpoint (HHP: s30, -3).

HHP “*no discrimination*, *the supervising physician has to adapt to the patient’s schedule and find appropriate times for appointments considering professional and family life*”.

HHP “[…] *I do not think such a trial will work for those patients* [diagnosed for more than 10 years]”.

#### ‘The moderately motivated viewpoints’: Rejection of side-effects: PLWH-VP3 and HHP-VP1

The PLWH-VP3 and the HHP-VP1 represented 15% and 19% of the total variability, respectively.

In contrast with the ‘*Reticence and way of life’* viewpoints, treatment and follow-up modalities were less important: constraints were accepted and personalization of follow-up was not expected (HHP: s6- -3; **s1**–2; s4–1; s5+ -1, s7 +1; PLWH: *s5*–2; s2- -1; s6, 1; s1- 0). These moderately motivated viewpoints were mainly characterized by their massive rejection of side-effects (HHP: **s9+**, **+4**; s10, +4; s8, +3; **s11+, +1**; PLWH: s9, +4; **s10**+ **+4**; s8+, +2; s11+, 0). Although the uncertainty associated with side-effects reinforced barriers to participation for the PLWH viewpoint (**s16+**, **+1**), this was not the case for the HHP viewpoint (s16-, -2).

PLWH “*HIV already damages organs a lot over time (all is not yet known on that point)*, *ART also …*. *Risks are increased only by the fact of being S+*. *I do not wish to add any other impact on vital organs”*.

HHP *“Primum non nocere = code of conduct*. *In the context of existence of efficient and well tolerated treatments”*.

PLWH *“It’s my first thought*. *It’s elementary common sense*. *Even greater importance was attached to the need to regularly and carefully take the treatment to avoid the risk of resistance*, *mutation*, *etc*. *It’d be difficult therefore not to think about it*, *in the framework of a trial where we would have to suspend the treatment and deal with the expected risks”*.

HHP *“not justified to bring on intense side-effects if the patient has a well-tolerated ART*, *since we do not know if the treatment stop will be beneficial to him”*.

For the two viewpoints the disease could not been forgotten (s24, PLWH: -3; HHP-: -2). HCRCT were seen rather as a means to avoid the long term side-effects of ART (s26, PLWH: +3; HHP: +2). Altruistic benefits were underlined (*s22*, PLWH: +3; HHP: +1; s23 *PLWH*: *+3*, HP: +2) and considered at least as important as personal benefits (s25-, PLWH/HHP: -3)

PLWH “*Personally*, *I do not find the current treatment constraining*. *One pill a day without particular side-effects*. *In principle I don’t mind at all taking it for the rest of my life*. *But really the only disadvantage of those years of regular exposure to medication is that they could have side-effects in the long term*, *on the liver*, *etc*. *And that’s why it’s important to find one day*, *if possible*, *a definitive or quasi-definitive curative treatment”*.PLWH “*several benefits*: *altruism*, *being an actor in the disease*, *taking things further; globally*, *for me*, *indirect benefits are as important as direct benefits”*.

Considering the trial to be more suitable for people who find it difficult to take ART on a daily basis was a common trait in both viewpoints, explaining in part their moderate motivation to participate (**s32+**, PLWH: **+2**; HHP: **+3**), and which is coherent with the emphasis on well tolerated ART. Although the PLWH viewpoint was not concerned with the selection of the target population, for the HHP viewpoint, no priority should be given to people with diagnosed HIV for more than ten years (**s30-, -4**) or those who have previously participated in only a few clinical trials (*s31*, -4) but with different reasons as illustrated below:

HHP “*I don’t see why the most recent patients should be excluded from these trials*. *I would even say that the people who’ve had the infection the longest and who experienced a succession of treatments with varying degrees of efficacy*, *would not have the best profile*”.

HHP “*I don’t see why the people having already contributed* [to research] *should be excluded from continuing this investment*. *Moreover*, *if they wanted to take part again*, *they would probably have a profile which is adapted to the trial’s requirements”*.

The PLWH viewpoint gave greater importance to research for a curative treatment than to access to ART for everyone (**s18-**, **-4**) and to prevention (**s19-**, **-3**). This could be another indication of the reason why this PLWH viewpoint was moderately motivated to participate in HCRCT, considering that it is too early at this point.

#### ‘The most motivated viewpoints’: PLWH-VP2 and HHP-VP2

The viewpoints PLWH-VP2 and HP-VP2 represented 16% and 15% of the total variability, respectively.

Their main common characteristic was the high level of acceptance of constraints, risk and side-effects compared with the six other viewpoints, as well as their acceptability of an ART-free duration < six months (s21+; PLWH/HHP +2). This acceptability was even greater for the PLWH viewpoint (**s4-**, **-4**; s7, -3; **s12-**, **-3**; **s8-**, **-2**; **s6-**, **-2**; *s5*, -2; s9-, -1; s11, -1; s10-, 0) than for the HHP viewpoint (s5, -2; s11, -2, s4, -1; s7, -1; **s10-**, **-1**; s9-, 0) for which the intensity of side-effects was a barrier to proposing HCRCT (s8, +3).

PLWH “*Way of life will necessarily be modified according to the intensity of treatments*, *consultations*, *hospitalizations; it’s integrally linked with research and I have to organise myself*, *…*, *it’s a choice*”.

PLWH “*I’ll participate even if the follow-up of the treatment-free period is very constraining because the stakes seem higher than transitory disadvantages”*.

For the HHP viewpoint—the most averse to uncertainty about side-effects (16+, +2)–particular emphasis was placed on the need for clear information and regular feedback from physicians and patients (**s28+**, **+4**; s29**+**, +4; s27+, +2). With respect to the PLWH viewpoint, the guarantee of the provision of information was also a motivation to participate but to a lesser extent (s27, +1; *s28*, +2, s29, +3), and uncertainty about side-effects was accepted (s16, -3). Moreover, this PLWH viewpoint was concerned about the personalisation of the follow-up (s1+, +2).

For these two viewpoints, the level of acceptance of side-effects and constraints was counterbalanced by expected benefits, particularly for the PLWH viewpoint. Indeed, these two viewpoints placed particular importance on the possibility of avoiding long term side-effects of ART (s26; PLWH**+: +**4; HHP: +3) and altruistic benefits (*s22*, PLWH**+,** +4; HHP: +3; *s23*, PLWH+3).

PLWH “*Treatments are not without impact on the organism*… *aging*, *pain*, *liver*! *I can manage but for how long*? *I have tamed the virus but treatment toxicity worries me*, *and I always hope to be free of it*… *taking the treatment makes you remember you’ve the virus each day”*.

PLWH “*I’m ready to move science forward*, *whatever the consequences*”.

Finally, this HHP viewpoint echoed PLWH-VP3, considering that investing in access to ART for the greatest number of PLWH (s18-, -3) and in prevention (s19-, -4) “*need not be in competition*” with investing in HRCT.

PLWH-VP2 was the PLWH viewpoint which placed most importance on the belief that participation in an HCRCT leads to improved quality of care (s17**+,** +1), and was the least opposed to financial compensation for participation being offered without it being requested (**s20+**, -1)

## Discussion

To improve future HCRCT designs, based on PLWH and HHP preferences and expectations, it is necessary to have a greater knowledge of the motivations for and barriers to participation in such trials, as well as an understanding of how the risks and the benefits of HCRCT are perceived. Our study contributes to the debate by investigating these issues. We complement recent literature [7,17,20,21,24] in two ways. First our surveyed sample not only included both PLWH who met the clinical criteria for inclusion in future HCRCT but also several HHP (nurses, physicians, research technicians) from different departments caring for PLWH. Some of the latter were not involved in HIV cure research. Second, we used a mixed method (Q_methodology) to elicit the structure of the main viewpoints of our respondents.

Five distinct viewpoints were elicited: two were population-specific (PLWH_VP1 and HHP-VP3), while the other three were composed of pairs of associated viewpoints from each population (Fig 2). A spectrum of viewpoints emerged ranging from the greatest motivation to participate (PLWH/HHP-VP2) to greatest reticence to participate (PLWH/HHP-VP4).

Interestingly, after performing the Q-sort exercise, 63% of the patients declared a willingness to participate in HCRCT, with an additional 34% saying ‘yes perhaps’ (equivalent percentages were obtained from HHP: 59% certainly, 34% perhaps). This result is in line with the HIV-specific culture of joint mobilization in the context of HIV research [2,22,43], and is consistent with previous quantitative surveys [7,17,21] where respondents mainly declared a motivation to participate in HCRCT [20]. When focusing on the specific viewpoints, with at least two (HHP-VP3 and PLWH/HHP-VP4) expressing reticence about HCRCT, our results are also consistent with qualitative surveys [24,44,20] where at least half of the respondents had little interest in HCRCT. This apparently contradictory result, also noticed by Dubé et al. [20], suggests more an effect of the method employed than opposition to the results. Furthermore, in line with psychosocial models, it highlights the need to consider all the determinants of any given behavior change [45], and the importance of not considering only declared intentions [29].

In our survey, as in others [7,17,20,21], altruistic benefits constituted a strong motivation for participating in HCRCT and direct clinical benefit was considered more important than altruistic benefits only for the *benefit-centred* HHP viewpoint (HHP-VP3). This result was reinforced in all viewpoints by the fact that financial compensation would not be a motivation to participate, and by the fact that HCRCT participation would be acceptable even with an ART-free period of less than six months, except again for the HHP *benefit-centred* viewpoint.

This result raises two differences with the literature. First, according to Arnold et al. [7], personal benefit (62%) is more important than social (56%) or scientific (45%) benefits for PLWH who consider participating in HCRCT. However, this distinction between personal, social and scientific benefits is not straightforward, not only because of the well-known altruistic value [46], but also because scientific advances may lead to future direct benefits for patients. Second, according to Dubé et al. [20], financial incentives have been found to be potential motivators. The difference in results might be related to the differences between the French and the US health systems, but also because the use of the Q-methodology allows prioritization between the dimensions at stake in the decision to participate/propose HCRCT, as discussed in the strengths and limitations section of this article.

In addition to altruism, the main motivation for participating/proposing HCRCT is the possibility of avoiding the long-term consequences of ART (for the *benefit-centred*, the *moderately-motivated* [PLWH-VP3/HHP-VP1] and the *most*-*motivated* [PLWH/HHP-VP2] viewpoints).

Another lever is the patient-physician relationship, with a consensus among PLWH and HHP that the patient’s HIV-physician needed to believe in HCRCT for patients and HHP to participate. Moreover, trial supervision by the HIV-physician was a motivation for participation for the *most-motivated* (PLWH-VP2) and *reticent* (PLWH-VP4) PLWH viewpoints, as well as for the *moderately*-*motivated* HHP viewpoint (HHP-VP1). As regards the patient-physician relationship, our results highlighted that physicians involved in HCRCT would need to provide regular feedback on the trial’s results as it progressed. This was particularly the case for the *most-motivated* HHP (HHP-VP2*)* and *reticent* PLWH viewpoints. Some viewpoints highlighted that similar feedback by participating patients was also expected, but to a lesser extent. This was especially true of the *most-motivated* HHP viewpoint. Clear information about HCRCT treatments was an essential prerequisite for nearly all viewpoints. This result has key implications both for the information to provide to patients in informed consent and for the management of the support during future trials, in particular regarding communication.

With respect to burden and support during the trial, the *reticent* viewpoint placed more importance on an unchanged way of life, and no loss of autonomy. Therefore, innovative treatment modalities and follow-up were considered barriers because of the resulting loss of autonomy. In contrast, regular and closely monitored follow-up during the ART free-phase was of great importance to the *most-motivated* and the *conditional-participation* PLWH viewpoints. This tension between the unavoidable medical consultations for the viral load tests during the period without ART treatment and the burden of participation in such trials have to be carefully managed by HHP.

Attitudes towards side-effects, and their impact on QoL, is a split line in the decision to participate/propose HCRCT. Any irreversibility of side-effects was a barrier to participation except for the *most-motivated* viewpoints. The possibility that vital organs might be affected by the innovative treatment, represented the second main barrier, for the PLWH *conditional-participation* [PLWH-VP1] and the *moderately-motivated* viewpoints. Globally, side-effects were only fully accepted by the *most-motivated* viewpoints, and conditionally by PLWH-VP1.Moreover, as has already been underlined in the literature [20], viewpoints reflecting uncertainty about side-effects, which is related to the need of information, was a barrier to participation for some viewpoints.

Finally, differences in opinion were highlighted between viewpoints regarding the possibility that participation in HCRCT would be specific to target populations. The *conditional participation* and the *most-motivated* PLWH viewpoints saw any participant selection based on criteria perceived as non-clinical, to be a barrier to equal access to care for all. This point mirrors [16], that the first HCRCT that might only recruit non-marginalised PLWH from industrialised nations, and would only be optimised for those nations’ health systems. The recruitment of a specific target population was also seen as a barrier to access to HCRCT for all by PLWH who inject drugs [24]. On the contrary, some viewpoints–which emphasized the fact that controlled patients had a quasi-normal life—considered HCRCT more suitable for patients who find it difficult to take ART on a daily basis.

With regard to the access to care, some viewpoints expressed a preference for developing prevention (*PLWH reticent-viewpoint*) and improving access to better medication for a higher number of PLWH (*HHP benefit-centred viewpoint*), rather than developing cure trials. This result reflects that by Chu et al. [44].

### Strengths and limitations

Q methodology, by enabling respondents to rank several statements, has the advantage of eliciting the synergies between the different dimensions explored while distinguishing more weighted statements into a common set of statements [38]. For example, although participants expressed that the risk of virus transmission during HCRCT was a major concern during the qualitative part of our study [41] as well as in the qualitative survey from Dubé et al. [20], Q-methodology helped reveal that this risk would no longer be a barrier to participation in HCRCT if evaluated in relation to the other statements. For some HHP it would mitigate but not block completely their motivation to propose the trial.

Our survey has limitations. First, no standard “cure” trial currently exists, and the envisaged trials are linked to medical advances. Indeed, although potential strategies have already been identified, as stated by Deeks et al., the challenges remains substantial [47]. As a result, the type of HCRCT described in our survey can be criticized, in particular given the impact of the word “cure” [19] and limits the generalizability of our results. However, it was designed according to the desires of participants during the qualitative step, who expressed the need for a description as least vague as possible, i.e. not just talking about “risk of side-effects” but describing their impact and their length. A need also highlighted by Arnold et al. [7]. Moreover, the Q-sort exercise provided additional information to the initial description of the trial about its potential effects and treatment administration.

Second, whilst Q studies are effective for eliciting and describing shared viewpoints on a subject, they are not a means for generating information about the proportion of the population who agree with these viewpoints [40,48]. To do this, we will provide a summarised description of the viewpoints during the next step of the survey [49].

## Conclusion

HIV cure research clinical trials (HCRCT) constitute a risky yet unavoidable step towards the goal of finding a cure [22]. To ensure good acceptability, participation and adherence by PLWH and HHP, HCRCT must be designed to meet criteria which are as acceptable as possible to all. Participating in or proposing an HRCT implies agreement between patients and health professionals [29] and this suggests the need for additional direct stakeholder-centred research.

Our results showed that scientific altruism and providing clear information on treatments and the associated side-effects are dimensions which have a strong impact in the decision-making process on whether to participate in or to propose HCRCT. Furthermore, we found that it is fundamental that the current HIV physician believe in HCRCT and participate in them. On the contrary, disparities were found between viewpoints regarding the accepted level of side-effects and the burden associated with HCRT participation on daily life, questioning the best strategy to adopt: whether to design HCRCT according to the expectations of the highest number of PLWH and HHP or to design them according to the most motivated PLWH and HHP.

## Supporting information

S1 Fig Second order analysis. (TIF)

S1 Table Description of HIV healthcare provider statements. (PDF)

S2 Table Idealized Q-sorts for people living With HIV and HIV healthcare provider. (PDF)
