# Supplementary figures and images for: Patterns of patient and healthcare provider viewpoints regarding participation in HIV cure-related clinical trials. Findings from a multicentre French survey using Q methodology (ANRS-APSEC)
Source: PLoS One. 2017 Nov 2;12(11):e0187489. doi: 10.1371/journal.pone.0187489 (PMC5667862; doi:10.1371/journal.pone.0187489)

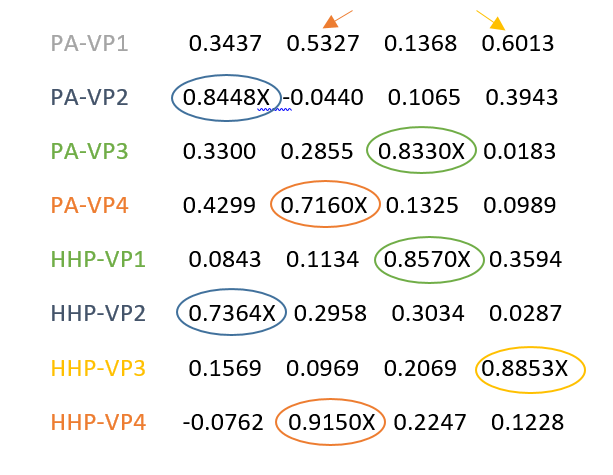

Supplement: S1 Fig — (TIF) [file pone.0187489.s001.tif]
