# Supplementary material for: Patterns of patient and healthcare provider viewpoints regarding participation in HIV cure-related clinical trials. Findings from a multicentre French survey using Q methodology (ANRS-APSEC)
Source: PLoS One. 2017 Nov 2;12(11):e0187489. doi: 10.1371/journal.pone.0187489 (PMC5667862; doi:10.1371/journal.pone.0187489)
Supplement: S1 Table — (PDF) [file pone.0187489.s002.pdf]

S1 Table. Description of HIV Healthcare Provider statements

**Treatment and follow-up**	s1	In order for me to propose this trial, the monitoring of the treatment-free phase must be adapted fully to each patient (i.e., tailor-made)
	s2	In order for me to propose this trial, the follow-up during the treatment-free phase must be regular and closely monitored (from once a week to once a day).
	s4	I would not propose this trial if the medical follow-up of the treatment-free phase were too restrictive
	s5	I would refuse to propose this trial if the innovative treatment were to last more than 6 months
	s6	I would only propose this trial if the innovative treatment were administered on an outpatient basis and did not require hospitalization
**Risks, Adverse effects and QOL**	s7	I would not propose this trial because of the possible increased risk of HIV transmission during the treatment-free phase
	s8	I would not propose this trial if the adverse effects of the innovative treatment were too acute (loss of autonomy or bedridden for 4 to 8 days per month)
	s9	I would not propose this trial if the innovative treatment were to affect vital organs
	s10	In order for me to propose this trial, the innovative treatment must not entail anything irreversible in terms of adverse effects
	s11	If the adverse effects were to last more than 5 days after taking the innovative treatment I would not propose this trial
	s12	In order for me to propose this trial, it is fundamental that the patient’s lifestyle does not change
**Physician-patient Relationship**	s3	In order for me to propose this trial, it is fundamental that the HIV patient’s treating physician supervise the three phases of the trial
	s13	In order for me to propose this trial, it is fundamental that the HIV patient’s treating physician believe in it
	s14	In order for me to propose this trial, a medical contact must always be available by phone
**Beliefs and Attitudes**	s15	I don’t believe in this type of trial
	s17	I believe that participating in a clinical trial guarantees better treatment
	s18	I think that it would be better to invest in access to ARV treatment for everyone
	s19	I think that it would be better to invest in prevention

**Benefits**	s20	Financial compensation for the patient could entice me to propose this trial
	s21	I would propose this trial if the treatment-free phase did not last more than 6 months
	s22	It is important to participate in HIV research
	s23	It is important that medical advances for future generations be made possible
	s24	One motivation to propose this trial would be that the patient could forget about the disease
	s25	Proposing a clinical (research) trial with no direct benefit for the patient has no sense
	s26	This trial could be a way to avoid the long-term consequences of ARV treatment.
**Information**	s16	There is too much uncertainty about the adverse effects for me to propose this trial
	s27	Having regular feedback from participating patients would motivate me to propose this trial
	s28	Being provided with regular information by physicians about the trial's results would motivate me to propose this trial
	s29	In order for me to participate, it is necessary that I have clear information about the treatments
**Target Population**	s30	This trial is more suitable for people diagnosed more than ten years ago.
	s31	This trial should primarily focus on those who have participated in only a few trials to date
	s32	This trial is more suitable for people who find it difficult to take ARV treatment on a daily basis
	s33	This trial is more suitable for people who do not work
