# Supplementary material for: Patterns of patient and healthcare provider viewpoints regarding participation in HIV cure-related clinical trials. Findings from a multicentre French survey using Q methodology (ANRS-APSEC)
Source: PLoS One. 2017 Nov 2;12(11):e0187489. doi: 10.1371/journal.pone.0187489 (PMC5667862; doi:10.1371/journal.pone.0187489)
Supplement: S2 Table — (PDF) [file pone.0187489.s003.pdf]

S2 Table. Idealized Q-sorts for People Living With HIV and HIV Healthcare Provider

**Summarized statements**	**PLWH-VP1** Conditional participation	**PLWH-VP2** Most motivated	**PLWH-VP3** Moderately motivated	**PLWH-VP4** Reticent	**Nb**	**HHP-VP1** Moderately motivated	**HHP-VP2** Most motivated	**HHP-VP3** Benefit-centered	**HHP-VP4** Reticent
trt-free phase monitoring must be adapted to each patient	1	2	0	1	1	-2	0	-4	1
trt-free phase follow-up must be regular and closely monitored	2	1	-1	0	2	0	1	2	-3
I would not participate if the medical follow-up of the trt-free phase were too restrictive	**-2**	-4	1	2	4	-1	-1	-2	2
I would refuse to participate if the innovative trt lasted more than 6 months	*-2*	*-2*	*-2*	*-2*	*5*	-1	-2	-2	-4
I would participate only if the innovative trt were administered on an outpatient basis	2	-2	-1	1	6	-3	0	-1	3
I would not participate because of the increased risk of HIV transmission during the trt-free phase	-1	-3	-2	-3	7	1	-1	-1	2
I would not participate if adverse effects of the innovative trt were too acute	**-1**	-2	2	1	8	3	3	0	4
I would not participate if the innovative trt affected vital organs	4	-1	4	0	9	4	0	1	1
The innovative trt must not entail irreversible adverse effects	**3**	0	4	1	10	4	-1	4	3
I would not participate if adverse effects lasted more than 5 days after the innovative trt	-1	-1	0	-3	11	1	-2	-2	-2

It is fundamental that my lifestyle does not change	1	-3	1	2	12	0	1	-3	3
It is fundamental that my current physician for HIV would supervise the three phases of the trial	*0*	*2*	*0*	*2*	*3*	2	-1	1	0
It is fundamental that my current physician for HIV would believe in the trial	*4*	*3*	*2*	*3*	*13*	*2*	*2*	*2*	*2*
A medical contact must always be available by phone	*0*	*1*	*-1*	*-1*	*14*	1	0	1	-1
I don’t believe in this type of trial.	-4	-4	-4	-1	15	-3	-4	-3	-1
I believe that participating in a clinical trial guarantees better care	**-2**	1	0	0	17	*0*	*-1*	*-1*	*-1*
I think that it would be better to invest in access to ART for everyone	0	0	-4	0	18	-2	-3	2	0
I think that it would be better to invest in prevention	-1	-2	-3	2	19	-1	-4	0	-2
Financial compensation could entice me to participate	-3	**-1**	-2	-4	20	0	0	0	-4
I would participate in this trial even if the trt-free phase did not last more than 6 months	0	2	1	1	21	1	2	-3	0
It is important to participate in HIV research	*3*	*4*	*3*	*3*	*22*	*1*	*3*	*3*	*1*
It is important that medical advances for future generations be made possible	*2*	*3*	*3*	*4*	*23*	2	1	2	4
One motivation for participation is that one could forget about the disease	**2**	**0**	-3	-4	24	-2	1	-1	-1
Proposing a clinical (research) trial with no direct benefit for the patient has no sense	**1**	**-1**	-3	-2	25	-3	-2	4	2

Participating in this trial could be a way to avoid the long-term consequences of ART	0	4	3	0	26	2	3	3	-1
There is too much uncertainty about the adverse effects for me to participate	-3	-3	1	-2	16	-2	2	1	0
Having regular feedback from participating patients would motivate me	1	1	1	-1	27	-1	2	0	1
Regular information by physicians about the trial's results would motivate me	*1*	*2*	*2*	*3*	*28*	0	4	1	0
It is necessary that I have clear information about the trt.	3	3	-1	4	29	3	4	3	1
This trial is more suitable for people diagnosed more than ten years ago.	**-4**	0	-1	-1	30	-4	1	-2	-3
This trial should focus on those who have participated in only a few trials before	-2	0	-2	-1	31	*-4*	*-3*	*-4*	*-2*
This trial is more suitable for people who find it difficult to take ART on a daily basis	-3	-1	2	-2	32	3	-3	-1	-2
This trial is more suitable for people who do not work	-1	1	0	-3	33	-1	-2	0	-3

Trt= treatment
